# Investigating the associations between upper limb motor function and cognitive impairment: a scoping review

**DOI:** 10.1007/s11357-023-00844-z

**Published:** 2023-06-20

**Authors:** Kaylee D. Rudd, Katherine Lawler, Michele L. Callisaya, Jane Alty

**Affiliations:** 1 Wicking Dementia Research and Education Centre, University of Tasmania, Hobart, Tasmania Australia; 2 School of Allied Health, Human Services and Sport, La Trobe University, Melbourne, Victoria Australia; 3 Menzies Institute for Medical Research, University of Tasmania, Hobart, Tasmania Australia; 4 Peninsula Clinical School, Monash University, Melbourne, Victoria Australia; 5 School of Medicine, University of Tasmania, Hobart, Tasmania Australia; 6 Neurology Department, Royal Hobart Hospital, Hobart, Tasmania Australia

**Keywords:** Upper limb, Motor function, Cognitive impairment, Dementia, Mild cognitive impairment

## Abstract

Upper limb motor function is a potential new biomarker of cognitive impairment and may aid discrimination from healthy ageing. However, it remains unclear which assessments to use. This study aimed to explore what methods have been used and to describe associations between upper limb function and cognitive impairment. A scoping review was conducted using PubMed, CINAHL and Web of Science. A systematic search was undertaken, including synonyms for key concepts ‘upper limb’, ‘motor function’ and ‘cognitive impairment’. Selection criteria included tests of upper limb motor function and impaired cognition in adults. Analysis was by narrative synthesis. Sixty papers published between 1998 and 2022, comprising 41,800 participants, were included. The most common assessment tasks were finger tapping, Purdue Pegboard Test and functional tasks such as writing. Protocols were diverse in terms of equipment used and recording duration. Most participants were recruited from clinical settings. Alzheimer’s Disease was the most common cause of cognitive impairment. Results were mixed but, generally, slower speed, more errors, and greater variability in upper limb movement variables was associated with cognitive impairment. This review maps the upper limb motor function assessments used and summarises the available evidence on how these associate with cognitive impairment. It identifies research gaps and may help guide protocols for future research. There is potential for upper limb motor function to be used in assessments of cognitive impairment.

## Introduction

The underlying brain pathology for most types of dementia develops over decades, prior to the cognitive symptoms emerging [1]. Motor changes related to this neuropathology have shown potential as non-invasive biomarkers [1–3]. In 2020, The 5th Canadian Consensus Conference on Diagnosis and Treatment of Dementia (CCCDTD) recommended that assessment of motor function should be included in dementia investigations as there is strong evidence that it can aid detection of cognitive impairment or dementia risk in older adults [2]. Motor biomarkers provide a low cost and accessible method for identifying early-stage cognitive impairment [4] and predict transition from mild cognitive impairment (MCI) to dementia [3, 4]. This may facilitate referral to specialised clinics, early risk modification and recruitment to intervention trials [5–7].

Gait has been the most studied motor biomarker with strong evidence showing cognitive impairment associates with impairments in gait [1, 5, 8, 9]. From gait studies, we know that the premotor cortex plays a key role in controlling and coordinating the neural activity in areas of the brain (such as the basal ganglia, brainstem and cerebellum) that are involved in planning and execution of movement [10]. The higher level control of the prefrontal cortex is further implicated when a cognitive task is performed while walking (dual-task). Damage to the prefrontal cortex caused by stroke or neurodegenerative disease is associated with gait impairment such as slowed walking speed and greater step time variability [2, 10–12]. Although the neurocognitive mechanisms underpinning the upper limb motor function (ULMF) changes seen with cognitive impairment are not fully understood yet, it would seem likely that they are comparable to those for gait.

Assessment of ULMF may provide additional benefits as many subtle measures of gait are undetectable by clinical observation and require electronic gait analysis systems which limits widespread access [13, 14]. In addition, gait analysis poses challenges for remote assessment and in people who have ambulatory difficulties. In contrast, analysis of ULMF is generally more accessible as it can be assessed using readily available mobile phones and computers and tests can be performed seated.

Emerging evidence shows that a range of ULMFs change in cognitive impairment and may aid discrimination from healthy ageing, but this has been less explored than gait [15–20]. It remains unclear what tasks of ULMF to use, how best to measure these and what movement variables associate with cognitive impairment. This hinders integration of ULMF assessments into investigations of cognitive impairment. This review thus aimed to address the question: ‘What *methods* of assessing ULMF have been used to investigate the association of ULMF with cognitive impairment in adults?’ via four sub questions:

1. What *tests* (including the task, equipment, protocol, and movement variables) of ULMF have been used to investigate cognitive impairment in adults?
2. What *conditions/diseases* with resultant cognitive impairment have been studied?
3. What were the major participant recruitment *settings*?
4. How does ULMF associate with cognitive impairment?

## Methods

A scoping review was conducted using JBI methodology for Scoping Reviews and reported following the Preferred Reporting Items for Systematic Reviews and Meta-Analyses—extension for scoping reviews (PRISMA-ScR) guidelines [21, 22]. A protocol was designed to define the questions and clarify methods and reporting (published in Figshare [23]).

We searched PubMed, CINAHL and Web of Science databases for studies published in English up to March 2022. Search terms included synonyms for the three main concepts: 1. Cognitive impairment, 2. Upper limb, 3. Motor function. We included terms describing specialised tasks of the hands and upper limbs such as writing, drawing and grasping. Appendix 1 shows the keywords and Medical Subject Headings (MeSH) terms used for the PubMed search.

## Eligibility criteria

All human research studies and systematic reviews examining the association between ULMF and cognitive impairment (caused by any disease/condition) in adults (≥ 18 years) were included. Books, theses, research protocols, and blogs were excluded. Eligible studies required at least one test of cognition and inclusion of participants with cognitive impairment. All tests involving dynamic and volitional functions of the upper limb were eligible, except for grip strength. Evidence from many other studies shows grip strength is associated with cognitive impairment and its measurement is recommended by the fifth Canadian Consensus Conference on Diagnosis and Treatment of Dementia (CCCDTD5) on early non-cognitive markers of dementia [2]. Furthermore, it could be argued that grip strength is not kinematic/dynamic, but rather a kinetic/isometric contraction. Studies of movement analysis in sleep were also excluded as these movements are considered involuntary. All methods of assessing ULMF were eligible.

## Data extraction process

Search results were exported to Covidence software, and duplicates removed. Titles and abstracts were screened independently by two reviewers (KR plus JA, MC or KL) against the eligibility criteria. Full texts of selected articles were retrieved and independently screened by two reviewers for inclusion; disagreements were resolved through consensus of the two reviewers and, when required, a third reviewer. As recommended by JBI manual for evidence synthesis [22], a draft data extraction table was developed, piloted, and revised by all authors before it was created in Covidence. This extraction table structured the researchers approach to ensure they extracted the same sets of data from each study and provided a logical summary of results based on the questions of the scoping review [22]. One author (KR) used this to extract data on each study’s design, recruitment setting and characteristics, disease or condition resulting in cognitive impairment, tests (including ULMF task, equipment, protocol, and movement variables) and key findings.

## Results

### Selection of sources of evidence

**Fig. 1:**
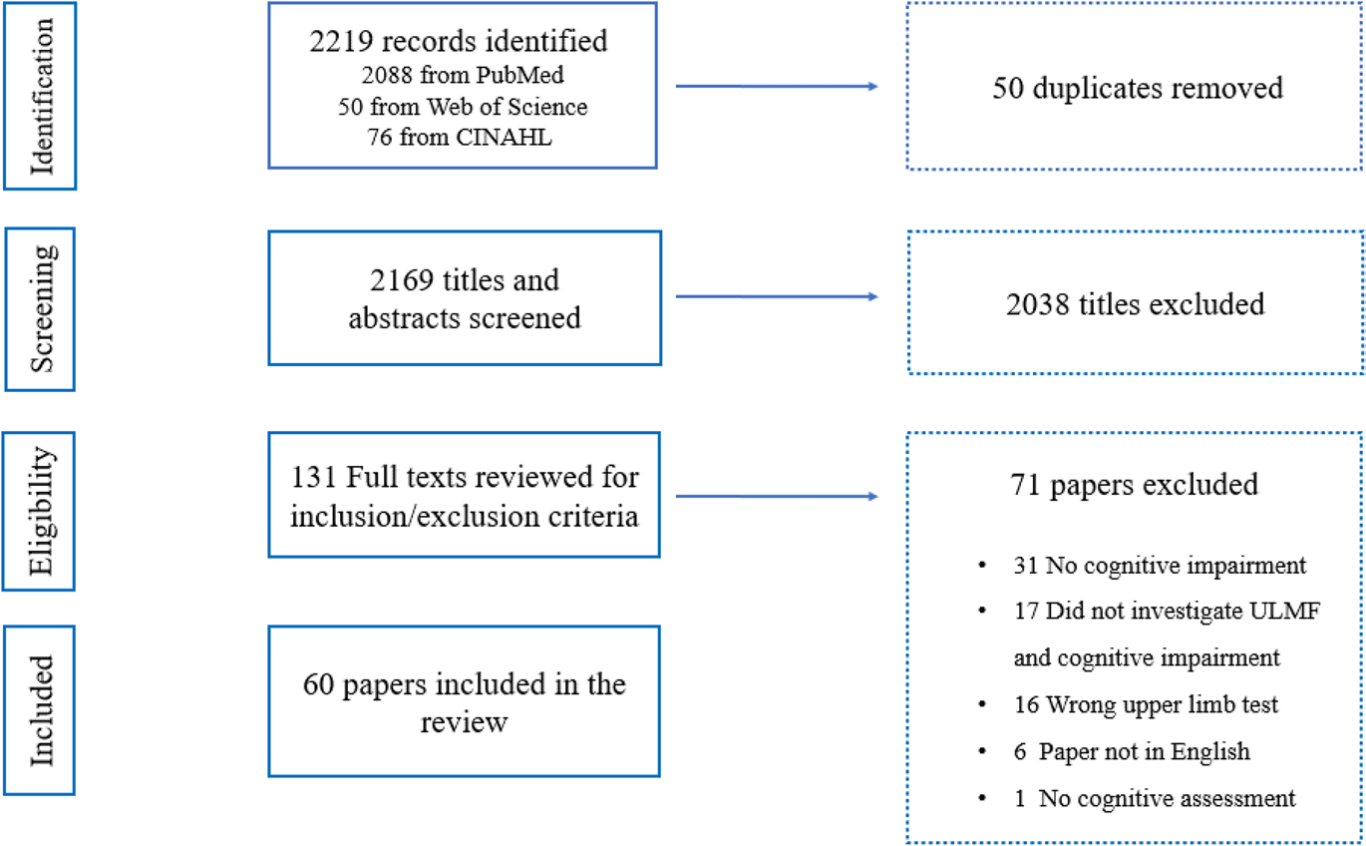
PRISMA flow diagram of the study selection in the review

2,219 records were initially identified and, after removing duplicates, 2,169 sources remained. Sixty papers met all selection criteria. Figure 1 shows the flow of information through the steps of this review.

### Characteristics of the evidence

Table 1 summarises the characteristics of 60 included articles. The papers were dated from 1995 to February 2022 and comprised 41,800 participants. Most studies (55%) were conducted in the United States, Germany, Japan, China, and the United Kingdom. There were 54 cross-sectional studies (90%), five longitudinal studies (8%) and one systematic review. Five of the cross-sectional studies were sub-studies of longitudinal cohorts.

- Sub-question 1. What *tests* of ULMF have been used to investigate cognitive impairment in adults?

**Table 1:** Characteristics of included papers

Author (year)	Country	Setting	Design	Population	Test(s) of cognition
Ott (1995) [24]	USA	Clinic	Cross-sectional	25 AD 25 HC	MMSE
Welch (1997) [25]	USA	Clinic	Cross-sectional	42 with KS 14 alcohol dependents without KS 26 HC	Wechsler Memory Scale-Revised
Camicioli (1998) [26]	USA	Community	Longitudinal (minimum 1.2 years)	85 at baseline: - 18 developed CI - 67 remained cognitively intact	Clinical Dementia Rating Scale
Goldman (1998) [27]	USA	Research	Cross-sectional	58 PD without dementia 22 PD with questionable dementia 43 HC	Comprehensive neuropsychological assessment
Willis (1998) [28]	USA	Clinic	Cross-sectional	26 AD 42 HC	MMSE
Goldman (1999) [29]	USA	Clinic	Cross-sectional	60 mild AD 43 HC	Wechsler Memory Scale
Schroter (2003) [30]	Germany	Clinic	Cross-sectional	35 AD 39 MCI 39 Major depression 40 HC	MMSE
Amieva (2004) [31]	France	Clinic	Longitudinal (2 years); a sub-study of a multicentre double-blind RCT	90 MCI at baseline: - 29 progressed to dementia - 61 remained dementia free	MMSE
Muhlack (2006) [32]	Germany	Clinic	Cross-sectional	12 AD 12 MCI 12 HC	MMSE
Bramell-Risberg (2010) [33]	Sweden	Community	Cross-sectional (part of a longitudinal study—Good Ageing in Skane)	301 CI 419 intermediate CI 1,207 HC	MMSE (grouped based on 3-word recall test)
Buracchio (2010) [6]	USA	Community	Longitudinal (mean 9 years)	204 at baseline: - 95 converted to MCI - 109 remained cognitively normal	MMSE
Ameli (2011) [34]	Germany	Clinic	Cross-sectional	8 MCI 8 AD	Comprehensive neuropsychological assessment
Rousseaux (2012) [35]	France	Clinic	Cross-sectional	31 AD 38 HC	MMSE
Rabinowitz (2014) [36]	Israel	Community	Cross-sectional	170 participants: - 97 with CI, - 73 without CI	MMSE
Henley (2014) [37]	UK	Clinic	Cross-sectional	20 bvFTD, 11 semantic PPA 4 non-fluent PPA 8 AD 31 HC	Comprehensive neuropsychological assessment
Johnen (2015) [38]	Germany	Clinic	Cross-sectional	20 AD 20 bvFTD 20 HC	MMSE
Ward (2015) [39]	Brazil	Clinic	Cross-sectional	52 AD 45 MCI 39 HC	MMSE
Nagahama (2015) [40]	Japan	Clinic	Cross-sectional	74 DLB 100 AD 52 VaD 75 HC	MMSE
Lin (2016) [41]	China	Clinic	Cross-sectional	10 AD 10 HC	MMSE
Toosizadeh (2016) [42]	USA	Clinic	Cross-sectional	10 CI 57 HC	MMSE MoCA
Fritz (2016) [43]	USA	Clinic	Cross-sectional	21 LBD 21 AD 21 PD 11 DLB 10 PDD	MMSE
Souza (2016) [44]	Brazil	Clinic	Cross-sectional	41 PD-AD 19 PD-MCI 41 PD 88 HC	MMSE
Dahdal (2016) [45]	Switzerland	Clinic	Cross-sectional	20 PD-MCI 31 PD-cognitively normal	MMSE
Darweesh (2017) [19]	Netherlands	Community	Longitudinal (Median 9.2 years) A sub-study of a prospective population-based Rotterdam Study	4856 at baseline: - 227 developed dementia, - 50 developed parkinsonism	MMSE
Kay (2017) [46]	USA	Clinic	Cross sectional	24 aMCI 41 APOEε4 carriers HC 65 non-carriers HC	MMSE
Kueper (2017) [3]	Canada	NA	Systematic review	NA	NA
Bartoli (2017) [47]	Italy	Clinic	Cross-sectional	20 CI 20 HC	MMSE
Sanin (2017) [48]	Austria	Clinic	Cross-sectional	45 AD 38 MCI 50 HC	MMSE
Garre-Olmo (2017) [49]	Spain	Clinic	Cross-sectional	23 AD 12 MCI 17 HC	Cambridge Cognitive Examination Revised
Suzumura (2018) [50]	Japan	Clinic	Cross-sectional	31 AD 15 MCI 48 HC	MMSE
Roalf (2018) [51]	USA	Clinic	Cross-sectional	131 AD 46 PD 63 MCI 62 HC	MMSE
Gupta (2018) [52]	India	Clinic	Cross-sectional	90 alcohol abstinent patients	MMSE
Rycroft (2018) [53]	USA	Community	Cross-sectional (Part of the Boston Rehabilitative Impairment Study of the Elderly (RISE))	68 aMCI 15 naMCI 98 mdMCI 249 HC	Comprehensive neuropsychological assessment
Gulde (2018) [54]	Germany	Clinic	Cross-sectional	11 AD 15 HC	MMSE
Jeppesen Kragh (2018) [55]	Denmark	Clinic	Cross-sectional	17 AD 19 FTD 13 DLB 15 HC	MMSE ACE
Zhang (2018) [56]	China	Community	Cross-sectional	20 MCI/no Tai Chi 20 MCI/Tai Chi 30 HC/no Tia Chi 30 HC/Tai Chi	MoCA
Carment (2018) [17]	France	Clinic	Cross-sectional	11 CI HC groups: - 10 young adults - 8 middle-aged adults - 11 older adults	MMSE
Fadda (2019) [57]	Italy	Clinic	Cross sectional	10 DLB 10 HC	MMSE FAB
Toosizadeh (2019) [58]	USA	Clinic	Cross-sectional	22 AD 24 MCI 35 HC	MMSE MoCA
Mollica (2019) [59]	Spain	Clinic	Cross-sectional	15 AD/Amyloid β + 20 HC/Amyloid β + 37 HC/Amyloid β -	Comprehensive neuropsychological assessment
Tomita (2020) [60]	Japan	Community	Cross-sectional	60 CI 42 CH	MoCA
Bologna (2020) [61]	Italy	Clinic	Cross-sectional	20 mild to moderate AD 20 HC	Comprehensive neuropsychological assessment MMSE
Liou (2020) [62]	Taiwan	Clinic	Cross-sectional	11,935 mild dementia 20,883 moderate to severe dementia	FUNDES-Adult
San Martin-Valenzuela (2020) [63]	Spain	Clinic	Cross-sectional	28 MHE 38 without MHE	MMSE
Hesseberg (2020) [18]	Norway	Community	Cross-sectional (part of a 1-year longitudinal study)	38 dementia 60 MCI	MMSE
Ntracha (2020) [64]	Greece	Community	Cross-sectional	11 MCI 12 HC	MMSE
Ehsani (2020) [65]	USA	Community	Cross-sectional	16 early-stage AD 30 aMCI 35 HC	MMSE MoCA
Zhang (2021) [66]	China	Research	Cross-sectional	20 MCI 41 HC	MoCA
Paixao (2021) [67]	Portugal	Community	Cross-sectional	22 dementia (institutionalised) 28 dementia (community dwelling) 26 HC	ACE
Mancioppi (2021) [68]	France	Clinic	Cross-sectional	17 MCI 27 HC	MMSE
Nagahama (2021) [69]	Japan	Clinic	Cross-sectional	162 AD 103 DLB	MMSE
Uwa-Agbonikhena (2021) [70]	Ukraine	Clinic	Cross-sectional	86 participants 1-year post-stroke	MMSE MoCA
Beeri (2021) [71]	USA	Community	Longitudinal (mean 7.3 years)	1160 with no CI at baseline 166 developed AD	MMSE
Zhao (2021) [72]	China	Community	Cross-sectional	35 AD/no exercise habits 35 AD/exercise habits 35 HC/no exercise habits 35 HC/exercise habits	MMSE MoCA
Suzumura (2021) [73]	Japan	Clinic	Cross-sectional	44 AD 20 MCI	MMSE
Colella (2021) [74]	Italy	Clinic	Cross-sectional	14 aMCI 16 HC	MoCA
Cosgrove (2021) [75]	UK	Clinic	Cross-sectional	22 PD-normal cognition 23 PD-MCI 10 PD-Dementia 19 HC-normal cognition 10 HC-MCI	MoCA
Davoudi (2021) [76]	USA	Research	Cross-sectional	29 AD 27 VaD 175 HC	Comprehensive neuropsychological assessment MMSE
Kutz (2022) [77]	Germany	Research	Cross-sectional (part of the SENDA study)	66 MCI 80 pMCI 79 HC	MoCA
Schmidt (2022) [78]	Germany	Clinic	Cross-sectional	47 PD	MMSE

Papers are presented chronologically according to publication date. *MMSE* Mini Mental State Examination, *AD* Alzheimer’s Disease, *HC* healthy controls, *PPA* primary progressive aphasia, *CI* cognitive impairment, *RCT* Randomised Clinical Trial, *KS* Korsakoff’s Syndrome, *LBD* Lewy Body dementia, *PD* Parkinson’s Disease, *MCI* mild cognitive impairment, *CCT* cube copying test, *bvFTD* behavioural variant frontotemporal dementia, *DLB* dementia with Lewy body, *VaD* vascular dementia, *MoCA* Montreal Cognitive Assessment, *PDD* Parkinson’s disease with dementia, *PD-AD* Parkinson’s disease with Alzheimer’s Disease, *PD-MCI* Parkinson’s disease with mild cognitive impairment, *aMCI* amnestic MCI, *naMCI* non-amnestic MCI, *mdMCI* multi-domain MCI, *Amyloid β + * Amyloid Beta positive, *Amyloid β -* Amyloid Beta negative, *ACE* Addenbrooke’s Cognitive Examination, *FAB* Frontal Assessment Battery, *FTD* frontotemporal dementia, *FUNDES-Adult* Functional Disability Evaluation Scale-Adults, *MHE* Minimal Hepatic Encephalopathy, *pMCI* possible MCI, *SENDA study* Sensor-based systems for early detection of dementia, *NA* not applicable

**Fig. 2:**
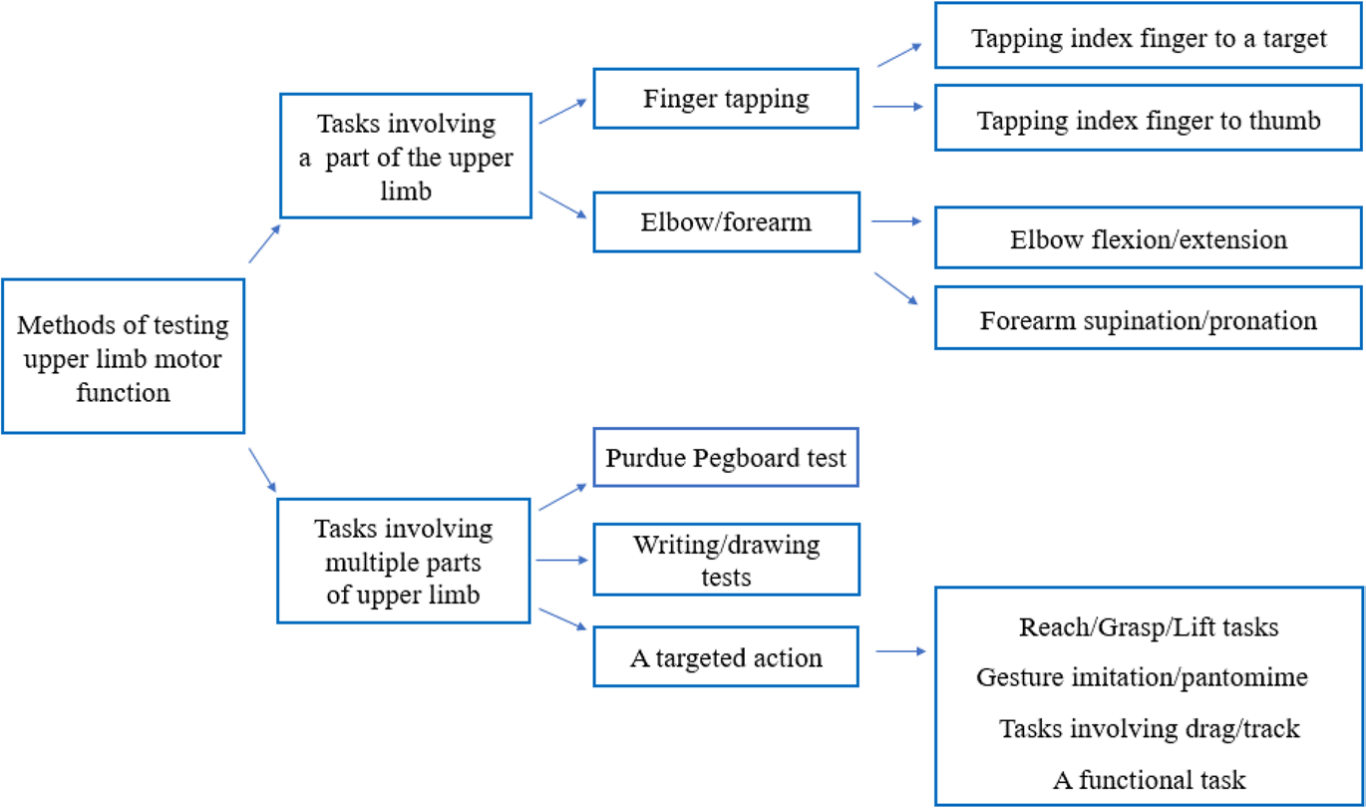
Task groups used for testing upper limb motor function. There were two major groups of tasks: 1) tasks involving a part of the upper limb, e.g., finger tapping or elbow flexion, and 2) tasks involving multiple parts of the upper limb, such as writing/drawing tests or Purdue Pegboard Test (PPT)

Table 2 and Fig. 2 outline the tests of ULMF in the included papers. The narrative synthesis considers the 4 main components of motor function tests: the task, equipment, protocol and movement variables. We recognise there are many ways to group upper limb assessments and that, some may argue that there are better ways to group the tests, especially for those that are new/experimental. In this review, and for ease of classification, we grouped the tasks based on the number of parts of the upper limb that are involved in completing the ULMF assessment.

**Table 2:** Summary of tests of upper limb motor function

Author (year)	Hand/upper limb assessed	Task	Equipment	Protocol	Movement variables
Ott (1995) [24]	Both hands	FT (index-target tapping)	Computer keyboard	Tapping any key of their choice at a fast pace for 8 s once with their index finger and then alternately tapped the index-middle finger	Number of taps (average of the five trials)
Welch (1997) [25]	Both hands	FT (index-target tapping)	Not specified	Finger tapping test as part of a neuropsychological battery (Halstead-Reitan manual) after 3 weeks of alcohol abstinence	Tapping speed Mean tapping score
Camicioli (1998) [26]	Both hands	FT (index-thumb tapping)	Not mentioned	Tapping index finger to thumb for 10 s (right and left hand)	Total number of taps for each hand
Goldman (1998) [27]	Both hands, dominant hand first	FT (index-target tapping)	An electronic device which is not specified	Tapping index finger for 10 s in 3 positions for each hand: wrist and elbow restrained, elbow restrained, and no restraint	Number of taps
Willis (1998) [28]	Both hands	1. FT (index-thumb tapping) 2. Forearm, supination/ pronation	Not specified	1. Tapping index fingers to thumbs at a fast pace for 10 s 2. Fast-paced supination/pronation of the dominant hand for 10 s	Number of correct cycles completed
Goldman (1999) [29]	Both hands, dominant hand first	FT (index-target tapping)	An electronic device which is not specified	Tapping index finger for 10 s in 3 positions for each hand: wrist and elbow restrained, elbow restrained, and no restraint	Number of taps
Schroter (2003) [30]	Dominant and non-dominant hand	Writing	Digitising tablet and a pressure-sensitive inking stylus	Drawing concentric circles on a digitising tablet as fast as possible for 30 s, then repeating the task while performing an additional distraction task with the nondominant hand for 10 s	Peak velocity SD of velocity Number of changes in direction
Amieva (2004) [31]	Not specified	FT (index-target tapping)	Computer keyboard	No details provided	Speed
Muhlack (2006) [32]	Both hands separately (dominant hand first)	PPT	25-hole computer-based contact pegboard	Transferring pegs from a rack into one of 25 holes in the board individually and as quickly as possible	Time taken to complete the task for each hand Total time
Bramell-Risberg (2010) [42	Both forearms	Forearm, supination/pronation	An optical shaft encoder (Hewlett Packard HEDS5701-A00) connected to a microcontroller (Microchip PIC 16C84) sending the data to the computer	Supinating and pronating each forearm separately for 10 s while gripping the handle of the shaft and bend their elbow approximately 90°	Number of supination/ pronation Speed
Buracchio (2010) [6]	Both hands (index fingers)	FT (index-target tapping)	A counting machine with a lever	Tapping a lever with an attached counter using the index finger of each hand for 10 s	Mean speed value of three trials
Ameli (2011) [34]	Dominant hand	Research/Grasp/Lift	A cylindrical and cordless object, mounted on top of an opaque plastic box which was either empty (400 g total) or contained an added 200 g mass (600 g total)	Lifting the object about 5 cm above the supporting table and holding for 4 s before putting it down under two conditions: with and without visual cues on weight of the object	Linear acceleration in three dimensions Peak acceleration
Rousseaux (2012) [35]	Both hands	Lille gestural apraxia test	Photos and various tools and objects as required	Imitating meaningless and symbolic gestures, pantomiming complex actions	Individual scores of each subtest Total score
Rabinowitz (2014) [36]	Dominant hand	FT (index-target tapping)	A touchpad mounted on a pressure transducer (FSR, InterLink Electronics, Camarillo, CA, USA)	Tapping index finger on a pressure pad for 15 s at a self-selected pace	Touch time Time off touch pad Touch cycle Touch SD Coefficient of variation
Henley (2014) [37]	Dominant hand	FT (index-target tapping)	Superlab on a computer	Tapping index finger on a computer key once in pace with the beat of a series of tones and once tapping in pace with a series of tones after the sounds ceased	Inter-response interval Inter-response interval variance Time variance Motor variance Response interval drift Response interval absolute drift
Johnen (2015) [38]	Both hands	The Cologne Apraxia Screening (CAS) Ideomotor Apraxia Test (IAT) The Monster Apraxia Items (MI)	Pictures of gestures	Pantomiming object use and commonly used gestures, and imitating hand and finger postures and face postures	Total score for each test Apraxia subdomain scores (% correct gestures)
Ward (2015) [39]	Dominant hand	Eight functional tasks from the Cambridge Cognitive Examination	Not specified	Drawings of pentagon, spiral, house, clock, and inserting a sheet of paper into an envelope, waving goodbye, cutting a sheet of paper with a pair of scissors, and brushing teeth	Sub-items scores Total score
Nagahama (2015) [40]	Both hands	Gesture imitation	NA	Imitating gestures after watching demonstration by examiner. Maximum 10 s was allowed to imitate each gesture	Accuracy scores Time taken to imitate each gesture Average scores of both hands
Lin (2016) [41]	Dominant hand	Trail making	A custom-made test device with a wooden board and 16 electronic plates with lights connected to a computer. A pencil-like stick was connected to the board by a wire	Striking lit target sensors with the pencil-like stick as fast as possible in three predetermined sequences: fixed pattern from left to right, random pattern and a fixed pattern while counting backward	Reaction time
Toosizadeh (2016) [42]	Dominant arm	Elbow flexion/extension	Wearable sensors: A tri-axial gyroscope and accelerometer sensor (BioSensics LLC, Boston, MA, USA)	Flexing and extending elbow at fast pace for 20 s. Then performed this test while counting numbers backward by one	Speed Flexibility Power Rise time Moment Speed reduction Speed variability Flexion number
Fritz (2016) [43]	Both (dominant then non-dominant hand)	PPT	9-hole Purdue Pegboard	Placing pegs into holes in a board and then removing them	Time
Souza (2016) [44]	Both hands	ILFT	NA	Imitating bimanual non-symbolic gestures after demonstration by examiner	Individual scores Total score
Dahdal (2016) [45]	Right hand	Functional tasks of upper limb such as holding the stylus inside a 5.8-mm-wide well for 20 s without touching the rim	An electric stylus	Holding the stylus and performing various tasks to test steadiness, precision, tapping, dexterity and aiming A total of 5 tasks were performed, each taking 20 s	Number of errors Number of taps Time to complete tasks
Darweesh (2017) [19]	Both hands (each hand separately and both hands together)	PPT	25-hole Purdue Pegboard	Placing as many cylindrical metal pegs as possible into one of 25 holes in a pegboard as possible in 30 s	Average score of three trials
Kay (2017) [46]	Right hand	FT (index-target tapping)	A box with a push button E-Prime software (Psychology Software Tools, Pittsburgh, PA)	Participants tapped a button with their right index finger for 12 s in synchrony with a visual flashing yellow and black checkerboard	Inter-tap intervals
Kueper (2017) [3]	NA	Finger tapping	NA	Not specified	Not specified
Bartoli (2017) [47]	Both hands (starting with the dominant hand)	Target tracking	A haptic interface (Omni®, Sensable) controlled by a custom-made software	Participants followed the target movement when it moved continuously on a computer screen, abruptly and when remained stationary. There were 22 trials, each lasting 20 s	Reaction times Mean absolute error with and without feedback
Sanin (2017) [48]	Both hands	Gesture imitation	NA	Imitating gestures/fingers configurations and hand movements	Individual scores of each test Total score
Garre-Olmo (2017) [49]	Dominant hand	Writing a sentence, drawing a clock face, and copying a house, pentagons, and a spiral	A commercial Intuos WACOM series 4 size L digitising tablet and a pressure sensitive Intuos ink pen	Writing sentences and drawing circles with a wireless electronic stylus on a paper fastened to the digitising tablet	Pressure Time Speed Acceleration
Suzumura (2018) [50]	Both hands	FT (index-target tapping)	JustTouch screen	Tapping marks on the screen using the index finger of right, left and then both hands for 15 s at a fast pace and in pace with sound signals	Mean lag time Lag-time SD Mean inter-tap time Intertap-time SD Contact time Contact time SD Inter-hand phase difference Inter-hand phase SD
Roalf (2018) [51]	Both hands	FT (index-target tapping)	Light Beam Finger and Foot Tapper Test (NeuroCognitive Engineering, 1995)	Tapping index finger through a light beam device for 10 s with dominant, non-dominant and then both hands. PD participants performed test while on medication	Total tap count Intra-individual variability Inter-tap interval
Gupta (2018) [52]	Both hands	FT (index-target tapping)	A custom-made software running on a laptop computer	Tapping index finger on a computer key for 10 s	Frequency
Rycroft (2018) [53]	Both forearms	Forearm, supination/pronation	NA	Supinating and pronating forearm of each arm separately for 20 s at a self-selected (two trials) and at a fast pace (two trials)	Number of accurate movements
Gulde (2018) [54]	Dominant hand	Gesture imitation	Pictures of objects	Imitating meaningless finger and hand gestures and performing pantomimes of object use	Correct imitations and pantomimes
Jeppesen Kragh (2018) [55]	Both hands (right and left hand individually)	1. FT (index-target tapping) 2. Forearm supination/ pronation 3. Reach/grasp/Lift	Force transducer (Mini-40, ATI Industrial Automation, Apex, NC, USA)	1. Tapping the force transducer for 10 s with the index finger at a fast pace, 2. Tapping the force transducer with the palm and back of their hands at a fast pace, 3. Grasping and lifting the force transducer and held it stable for 20 s at a height of 10 cm	1 & 2 Frequency Mean inter-onset interval Inter-onset interval SD 3 Mean orientation index Mean position index
Zhang (2018) [56]	Both hands	FT (index-target tapping)	An infrared photoelectric sensor connected to a computer	Placing index finger within the frame and tapping at a fast pace for 8 s	Frequency
Carment (2018) [14]	Dominant hand	FT (index-target tapping)	Finger Force Manipulandum; a device with force sensitive pistons	Matching the applied index finger force to the target force using the visual feedback and then using auditory cues	Frequency Variability
Fadda (2019) [57]	Both arms	Hand to Mouth movement	Wearable sensors: Markers were placed on upper limb bony landmarks. An 8-cameras motion-capture system (SMART-D, BTS Bioengineering, Italy)	Reaching, touching mouth and returning to initial position. Arms were tested separately	Time events (total and each phase) Speed, Smoothness Accuracy variables
Toosizadeh (2019) [58]	Dominant arm	Elbow flexion/extension	Wearable sensor: A tri-axial gyroscope and accelerometer sensor (BioSensics LLC, Boston, MA, USA)	Flexing/extending the elbow under two conditions: fast-paced for 20 s, and self-pace for 60 s. Self-selected pace was performed as a single-task and under two dual-tasks	Speed Rise time Flexion number Range of motion Variability
Mollica (2019) [59]	Both hands	FT (index-target tapping)	Computer keyboard (E-Prime 2.0 (Psychology Software Tools Inc., Pittsburgh, PA)	Tapping computer's spacebar with their index finger as fast as they could for 10 s while looking at a fixation point on the monitor. Hands were tested separately	Speed Intrasubject variability
Tomita (2020) [60]	Both hands	FT (index-target tapping)	Wearable sensors: Magnetic sensors were placed on the thumb and index finger of both hands. Magnetic sensors (UB-2; Hitachi Maxell, Tokyo, Japan)	Tapping index fingers to thumbs at a fast pace for 15 s once in-phase and once alternately Instruction: open fingers 5 cm while tapping	Amplitude (movement distance between the thumb and index finger) Total tap count Rhythm
Bologna (2020) [61]	Right hand	FT (index-thumb tapping)	Wearable sensors: Reflective markers attached to the tips of the index finger and thumb and detected via an optoelectronic system (SMART motion system, BTS Engineering, Italy)	Tapping index finger to thumb at a self-selected pace for 15 s	Amplitude Velocity Movement slope Rhythm
Liou (2020) [62]	Both hands	Domain 8 of FUNDES-Adult assessment	FUNDES-Adult assessment records	Assessment included pen-holding, buttoning, and knotting	Level of assistance required to complete each task
San Martin-Valenzuela (2020) [63]	Both hands	FT (index-target tapping)	Not specified	Tapping a key at a fast pace using the index finger of each hand separately and then both hands simultaneously for 10 s	Number of finger-taps
Hesseberg (2020) [18]	Dominant hand	FT (index to target tapping)	A counting machine	Tapping index finger of the dominant hand at a fast pace for 10 s on a counting machine	Mean tap number of the five trials
Ntracha (2020) [64]	Not specified	Typing on a virtual keyboard	Custom-made virtual keyboard on an app on Smartphones	Typing down up to 4 short texts, around a paragraph in length about familiar topics. No time limit. Phones’ autocorrection feature were disabled	Number of errors Keystroke timing
Ehsani (2020) [65]	Dominant arm	Elbow flexion/extension	Wearable motion sensors (tri-axial gyroscope sensors (BioSensics LLC, Cambridge, MA)	Flexing/extending elbow at a self-selected pace for 60 s under one single-task and two dual-task conditions	Entropy Angular velocity
Zhang (2021) [66]	Dominant hand	Drag and Drop Test	A Huawei M5 touch screen tablet	Dragging blocks one by one from a start area and dropping them to a target area within 60 s without hitting a partition in between the start and target areas or dropping the blocks in the start area	Number of successful and failed dragged blocks Time taken to move a block Average speed Speed SD
Paixao (2021) [67]	Both hands	Grocery Shelving Task (putting grocery cans on a shelf)	Adjustable shelf and twenty 420-g grocery cans divided into two grocery bags	Standing up from a chair, walking 1 m toward the shelf, and placing all the cans in the shopping bags on the shelf as quickly as possible, one can in each hand	Time
Mancioppi (2021) [68]	Dominant hand	FT (index-target tapping)	Wearable sensors, based on microelectromechanical sensors composed by the SensHand	Tapping index finger at a self-selected pace for 15 s, under single- and dual-task conditions	Number of taps Dual-task cost Opening velocity Velocity SD
Nagahama (2021) [69]	Both hands	Gesture imitation	Not specified	Imitating gestures after watching examiner’s hand with each hand separately and then simultaneously	Time taken to imitate Accuracy of imitation
Uwa-Agbonikhena (2021) [70]	Both hands	Various tasks such as hook grasp, cylinder grasp, elbow flexion/extension, forearm supination and pronation, etc	The Fugl-Meyer assessment	As per Fugl-Meyer upper extremity assessment of sensorimotor function protocol	Subtest scores Total motor function score
Beeri (2021) [71]	Both hands	1. PPT 2. FT (index-target tapping)	1. 25-hole Purdue Pegboard, 2. an electronic tapper (Western Psychological Services, Los Angeles, CA)	1. Inserting as many pegs as possible in 30 s 2. Tapping the tapper with their index finger at a fast pace for 10 s	1. Average score of two trials for each hand 2. Average score of two trials for each hand (number of taps)
Zhao (2021) [72]	Both hands)	FT (index-target tapping)	An infrared photoelectric sensor	Tapping index finger for 8 s at a fast pace within the device's frame first with the right and then with the left hand	Frequency
Suzumura (2021) [73]	Both hands	FT (index-thumb tapping)	A FT device with magnetic sensors (UB-2, Maxell Holdings, Ltd, Tokyo, Japan) Colour-coded cables were attached to the dorsum of participants' index fingers and thumbs	Tapping index finger to thumb at a fast pace for 15 s under four conditions: a single hand at a time, both hands simultaneously and both hands alternately	36 variables such as: Max amplitude Max velocity SD of velocity Accretion SD of inter-tapping interval Inter-tapping interval variability
Colella (2021) [74]	Dominant hand	FT (index-thumb tapping)	Wearable sensors: An optoelectronic system (SMART motion, BTS Technology, Italy)	Tapping index finger on their thumb as widely as possible at a fast pace for 15 s in three trials	Amplitude Velocity Rhythm
Cosgrove (2021) [75]	Both hands (trials alternated between hands)	Reach/Grasp/Lift	The object was a cylindrical Philips Imageo rechargeable candle made of Perspex. Movements were recorded using a Polhemus Patriot electromagnetic tracking device	Grasping the object, lifting it vertically and then placing it back on the table. Five trials for each hand completed under four conditions: normal lighting (self- and fast-paced) and self-selected pace (darkened room and eyes closed)	Reaction time Movement time
Davoudi (2021) [76]	Dominant hand	Drawing (digital Clock Drawing Test)	Digital pen	Drawing a clockface with hands to 10 after 11 on paper using a digital pen	37 variables including: Number of strokes Completion time Velocity Average pen pressure on paper
Kutz (2022) [77]	Dominant hand (dominant index finger)	FT (index-target tapping)	A force transducer (manufacturer: Measurement Specialties Inc., Hampton, VA, USA; Model: FX-1901–0001-50 L)	Tapping index finger on the force transducer for 15 s and under two different conditions: at a self-selected pace (three trials) and at a fast pace (two trials)	Tapping cycle time and its components (time on the device and time off) Maximum tapping force
Schmidt (2022) [78]	Both hands	ILFT	Camera to take photos of gestures for subsequent evaluation	Imitating bimanual non-symbolic gestures after demonstration by examiner	Individual scores Total score

*FT* finger tapping; *PPT* Purdue Pegboard Test; *ILFT* Interlocking Finger Test, *SD* Standard Deviation

#### Tasks involving a part of the upper limb

##### Finger tapping

Finger tapping (FT) was the most common task with twenty-seven (45%) studies using it as the main task, or one of the tasks. Nearly all studies analysed FT frequency or number of finger-taps. Protocols have evolved with advances in technology, allowing more precise recording with more recent papers including analysis of additional variables such as time between taps and rhythm fluctuations. FT was performed either by tapping a key/lever with the index finger (index-target tapping) or tapping the index finger to the thumb (index-thumb tapping):

*Index-target tapping*

Twenty-two (37%) studies used this task. The first, published in 1995 [24], used a computer keyboard to count the number of fast-paced finger-taps. Since then, eight studies used fast-paced tapping of a computer key or lever [6, 18, 27, 29, 55, 59, 63, 71]. Movement variables such as number of taps and tapping speed were extracted. Three studies measured self-paced and fast-paced FT for 15 s, using a force transducer [36, 77] or touchpad [50]. Three studies employed ‘cued’ FT protocols requiring tapping to defined frequencies paced by auditory cues [51] or visual cues [46].

More recent studies used infrared-light sensor technologies to measure FT: two used photoelectric sensors arranged around a frame to measure fast-paced FT over 8 s [51] and one required participants to tap their index finger through an infrared light beam for 10 s [51]. Another study [68], used wearable electromechanical sensors on the index finger during 15 s of comfortable pace tapping on the table.

*Index-thumb tapping*

In total, five (8%) studies used index-thumb tapping; the first, published in 1998, measured the number of taps in 10 s performed by people with Alzheimer’s Disease (AD) [28]. In four recent papers (since 2020) participants were asked to tap at a fast pace for 15 s while wearing reflective markers or magnetic sensors on their thumb and index finger [60, 61, 73, 74]. Extracted movement variables included speed, amplitude, and variabilities in time and speed of a finger-tap cycle [60, 61, 73, 74].

##### Elbow/forearm movements

The first study of forearm movement in cognitive impairment was in1998 [28] and researchers visually counted the number of correct supination/pronation cycles in 10 s. The next study was 12 years later [33] using an optical shaft encoder to measure the number of fast-paced supination/pronation cycles in 10 s. Since then, two studies used wearable 3-D gyroscopes to measure additional variables such as speed, rise time and speed variability of self-paced and fast-paced elbow flexion movements in 20 s [42, 58] and one used the same device to assess speed and variability of self-paced elbow flexions over 60 s [65].

#### Tasks involving multiple parts of the upper limb

##### Purdue Pegboard Test (PPT)

The PPT involves placing a series of pegs into holes on a board as fast as possible and has been utilised in four studies. Three used the 25-hole PPT [19, 32, 71] and one used a 9-hole pegboard [43]. Studies used various protocols: two measured the number of pegs inserted into holes of a 25-hole pegboard in 30 s [19, 71] and two timed participants inserting pegs and removing them from a 25-hole pegboard [32] and a 9-hole pegboard respectively [43].

##### Writing/drawing tasks

Three studies [30, 49, 76] used writing or drawing tasks to investigate whether kinematic measures (such as speed and smoothness) of digital pen movements on a digitising tablet or paper could differentiate between people with cognitive impairment—including MCI, AD and Vascular dementia (VaD)—and healthy controls (HC). In one study [76], participants drew a clockface on paper using a digitising pen. In another, participants drew concentric circles on a digitising tablet, at a fast pace [30]. In a study [49] which included sentence writing tasks too, participants drew circles on a digitising tablet at a self-selected pace.

##### Reach/Grasp/Lift tasks

Three studies used tasks involving reach/grasp/lift of an object [34, 55, 75]. In one study [34], participants with MCI and AD lifted objects with different weights and held them for 4 s. In another [55], participants with dementia (various types) lifted an object for 20 s. Both studies analysed steadiness and speed. One study [75] assessed reaching for an object at self-selected and fast paces, under various visual conditions in HC and Parkinson’s Disease (PD), some with cognitive impairment, measuring the time to complete the task.

##### Gesture imitation

Six studies analysed the ability of participants to imitate bimanual hand gestures after watching a demonstration by the examiner and recorded the number of correct performances and number of errors [35, 38, 40, 48, 54, 69]. In one study, the examiner demonstrated gestures sitting next to the participants to reduce perceptual complexities [48]. In the rest, examiners demonstrated gestures in front of participants [44, 78]. Two studies used the Interlocking Finger Test (ILFT) [44, 78] in which the examiner demonstrates specific shapes with their hands one at a time, and then asks the participants to imitate those gestures, as accurately as possible—for example, interlocking the fingers in a particular manner.

##### Dragging or tracking tasks

One study used a robotic haptic interface to measure reaction times and mean error of tracking movements in participants with cognitive impairment [47]. The device guided the hand to a target position and gave real-time visual feedback about the hand position as it tracked the target.

Another study used a custom-made electronic board to measure target-tracking reaction times in AD and HC [41]. An electric pen has been used to measure tracking movement variables (such as number of errors and total time) of various upper limb tasks such as hitting targets or guiding the pen through a narrow space [45]. In a recent study, participants used a computer tablet to drag virtual blocks to a target without dropping them in the wrong area [66]; the number of successful and failed attempts within 60 s, and time taken to move a block were analysed.

##### Tasks resembling day-to-day upper limb functions

One study explored ULMF in people with dementia by measuring the time taken to shelve groceries [67]. Another analysed “hand to mouth” movement variables such as time, speed, and smoothness, in dementia with Lewy bodies [57]. One study used a smartphone app custom-made keyboard to analyse characteristics of virtual key presses (such as keystroke timing) during typing [64].

Three studies analysed participants’ movements as they followed a specific protocol of various functional tasks, and scores were given by observation of their performance. One used parts of the Cambridge Cognitive Examination involving tasks such as putting paper into an envelope, waving goodbye, cutting paper with scissors and brushing teeth [39]. One study used part of the Functional Disability Evaluation Scale-Adult version (FUNDES-Adult), which includes pen-holding, buttoning, and knotting tasks [62]. While another chose the Fugl-Meyer assessment [70].

- Sub-question 2. What *conditions/diseases* with resultant cognitive impairment have been studied?

Table 3 summarises the conditions or diseases leading to cognitive impairment included in this review. Of 54 cross-sectional studies, 37 included participants with dementia—26 of them with AD diagnosis—and 22 had a group with MCI. Eight studies investigated participants with PD and cognitive impairment, one recruited participants with Minimal Hepatic Encephalopathy [63]. Two studies recruited participants with cognitive decline related to excessive alcohol consumption [25, 52]. Participants with no known cognitive impairment were recruited in seven studies and after testing for cognitive impairment allocated to groups with and without cognitive impairment. Of five longitudinal studies, four recruited people with no known cognitive impairment at baseline [6, 19, 26, 71] and one recruited participants with MCI [31].

- Sub-question 3. What were the major recruitment *settings*?

**Table 3:** Diseases or conditions leading to cognitive impairment

Disease/conditions causing cognitive impairment	Number of papers
Alzheimer’s Disease	Twenty-six [21, 25, 27, 28, 30, 34, 36, 39, 43, 46, 48, 51, 52, 54, 55, 59, 60, 63, 65, 66, 68, 73, 75, 77, 78]
Parkinson’s Disease	Eight [24, 30, 31, 33, 37, 53, 73, 74]
Dementia with Lewy Bodies (DLB) or Lewy Body Dementia (LBD)	Four [28, 39, 69, 75]
Frontotemporal Dementia (FTD)	Three [46, 51, 75]
Alcohol or drug related	Two [28, 33]
Vascular Dementia (VaD)	Three [22, 49]
Minimal Hepatic Encephalopathy	One [41]
Stroke	One [62]
Depression	One [63]

Forty-two studies recruited participants from clinical settings (e.g., neurological/ memory/cognition clinics, hospitals or rehabilitation centres), fourteen from community settings such as primary health services, day centres and exercise classes [6, 18, 19, 26, 33, 36, 53, 56, 60, 64, 65, 67, 71, 72], and four from research settings (e.g., from other research cohorts) [27, 66, 76, 77].

- Sub-question 4. How does ULMF associate with cognitive impairment?

Most studies of index-target tapping found significant differences between HC and people with dementia [24, 25, 37, 51, 59, 71, 72] and MCI [6, 17, 26, 36, 46, 51, 56, 63, 77]. Generally, MCI and dementia were both associated with slower, less rhythmic and lower frequency finger-taps. However, two studies found no association between tapping frequency and cognitive impairment [27, 29].

Studies of index-thumb tapping had mixed results too. Three studies reported associations between cognitive impairment and lower frequency, and increased variability, of FT [26, 60, 73]. However, two studies using wearable sensors, found no differences between FT frequency and amplitude in MCI or AD compared to HC [61, 74] although one found FT in MCI was less rhythmic [74]. The systematic review [3] concluded that FT was not associated with incident dementia in people with MCI.

For studies of forearm supination/pronation, slower speed and increased variability were associated with cognitive impairment [28, 33, 53, 55]. The three studies of elbow flexion [42, 58, 65] found no differences under single-task condition between participants with cognitive impairment (MCI and AD) and HC but with dual-task conditions (elbow flexion and a cognitive task), there were significant associations.

All studies using the PPT found dementia was associated with slower movements compared to MCI [18, 19, 32, 43]. All studies analysing writing/drawing kinematics found increased irregularity of movements, variability in speed and decreased accuracy differentiated HC participants from AD [30, 39, 49, 76] and from MCI [30, 39, 49]. One study [76] that compared measures of clock drawing in AD and VaD found that VaD drew more slowly (having slower speed and taking longer to draw).

Using reach/grasp/lift tasks, one study [34] found no differences between those with MCI or dementia, but another found dementia was associated with more variability than MCI [18]. A study of PD reported that those with dementia [75] had longer reaching reaction times. Studies employing gestures found significant imitation impairment in participants with dementia [35, 40, 48, 54, 69]. The inability in correct imitation of gestures in the ILFT was also correlated with cognitive impairment [44, 78]. Studies using functional tasks found variables of ULMF (such as increased time to complete the task, decreased smoothness, and less accuracy of movements) correlated with cognitive impairment [45, 57, 62, 67, 70]. Studies measuring tracking abilities found that participants with MCI and AD had more errors and slower reaction times than HC [41, 47]. Using digital tests, two studies [64, 66] showed differences between MCI and HC: one [66] reported reduced speed of dragging virtual blocks and another [64] identified more errors in virtual keyboard presses.

Among the longitudinal studies, two [6, 26] found that slower baseline FT in HC was associated with cognitive impairment at follow up, but another [31] found no such association. One study [19] found lower PPT scores were associated with higher risk of developing dementia at follow up. A study [71], using both PPT and index-target tapping concluded that lower performance scores of both tests were associated with risk of MCI and dementia at follow up.

Fifteen studies additionally investigated how ULMF associates with individual cognitive domains. Eight studies, using index-target tapping, found significant associations between FT variables (tapping cycle time, tapping rate and time variability) and memory (working and episodic), verbal fluency and executive function [18, 24, 27, 36, 37, 46, 59, 63]. One study found slowed index-thumb tapping speed associated with verbal fluency and executive function but not with delayed memory [25].

One study of elbow flexion found associations between speed and rhythm fluctuations with executive function [42]. Lower PPT scores correlated with impaired attention, visuo-spatial and executive function [18]. Using gesture tasks, studies found imitation accuracy associated with verbal fluency, attention [44, 69, 78] and executive function [44] but another did not [78]. Tracking ability was correlated with memory and visuospatial domains [47] and functional tasks were associated with attention, visuospatial and executive function [70].

## Discussion

Sixty studies published between 1995 and 2022, and comprising 41,800 participants, met the criteria to inform this review. To our knowledge, this is the first review investigating the association of ULMF with cognitive impairment. The studies used a diverse range of ULMF tasks from a simple movement, such as FT or elbow flexion, to more complex movements such as writing/drawing. Studies also used a range of protocols (self-paced, fast-paced, dual-task etc.), test durations (ranging from 8 to 60 s), and equipment. With technology advancements over time, the precision of data collection equipment has progressed, so analyses have evolved from counting the number of repetitions to detailed quantification of rhythm, amplitude and speed. The recruitment settings were mostly clinical, and the conditions included were predominantly AD, MCI and PD. Many studies found that, compared to age-matched older adults, people with cognitive impairment had slower speeds, longer reaction times and more errors and variability in their ULMF performance. However, these associations were not universal, especially among the FT studies. FT (index-target or index-thumb tapping) was the most common ULMF test, but protocols, durations and equipment varied significantly among these studies which may be the reason why FT studies had mixed results. Studies of elbow flexion, writing/drawing tasks and the PPT had no conflicting results, although there were fewer studies compared to the large number that assessed FT.

With no limitation in dates, this review provides a broad view of how ULMF assessments in the context of cognitive impairment have evolved since conception about 25 years ago. We systematically searched published literature using established guidelines and published our protocol in advance in an open access repository (Figshare). It is important to acknowledge that we excluded studies measuring hands/arms strength, such as grip strength, and excluded studies with only healthy participants which may have excluded some of the ULMF tests. We also acknowledge that it is possible relevant studies without linked keywords may have inadvertently been excluded.

This review highlights that ULMF assessments hold potential to be used in cognitive impairment investigations as many (but not all) of the studies found associations between ULMF and cognitive impairment. However, it also revealed a major gap in the current literature and that is the lack of consistency between the experimental methods used to assess ULMF. It remains unclear whether one specific type of test is superior to others, and it remains unclear how many repetitions of a task, or what test duration, should be used to balance sensitivity with potential effects of fatigue. The review demonstrated that, in a similar way to how gait analysis now has some recommended standard protocols [14], there remains a need to also standardise ULMF assessment methods—in terms of test durations and protocols (fast-paced vs. self-selected pace); this would substantially aid comparison of studies and clarify which tests are most discriminatory.

Most ULMF studies used 10 to 15 s as the test duration which seems to be a pragmatic balance between capturing enough data for robust analysis of movements whilst minimising the effects of fatigue. Several studies measured ULMF performance at various paces (self-selected pace vs fast pace) or under different conditions (single task and dual task). These approaches, as well as analysing multiple component measures of movements (such as frequency, speed, amplitude and rhythm) appeared to be more sensitive to cognitive impairment than testing just one movement under one condition and/or few movement components. Future research should consider analysing frequency, speed, and variability of ULMF as the core measures as these have repeatedly been shown to associate with cognitive impairment.

As most studies of ULMF have compared healthy controls to just one group who had clinically-manifested cognitive impairment, especially Alzheimer’s Disease (AD) dementia, it remains unclear how ULMF changes across the dementia continuum. Furthermore, there have been relatively few studies of other types of dementia. Future research should therefore aim to recruit participants with earlier stages of dementia pathology, such as subjective cognitive impairment, MCI, and early-stage dementia to provide richer insights into the changes related to disease progression. It was noteworthy that most studies classified participants according to screening tool cut-off scores rather than a more comprehensive cognitive assessment using established diagnosis criteria; we would recommend that future researchers aim to ascertain a more rigorous evaluation of the various domains of cognitive impairment as this would allow a more granular comparison with ULMF features and the opportunity to explore whether certain underlying pathologies have specific ULMF motor signatures.

For ULMF assessment to be included in CCCDTD as a recommended motor function assessment in dementia investigations, it is necessary to know how best to assess ULMF that is significantly associated with cognitive impairment and dementia. We are still learning about the association between ULMF and cognitive impairment and methods of testing ULMF are yet to be fully explored. This review shows that despite some inconclusive results, there is emerging evidence to support including ULMF in cognitive impairment investigations.

## Conclusion

In this scoping review, we summarised the current available evidence on the association of ULMF and cognitive impairment and also the tests, protocols, recruitment settings and conditions used to assess this association. Of the identified methods of ULMF assessment, FT was the most commonly used test followed by functional tasks of upper limb, PPT and elbow/forearm movement. Despite some mixed results, the ULMF movement variables were generally associated with cognitive impairment and could aid in distinguishing cognitive impairment from healthy ageing.

## Appendix 1

**Table:** Key search terms for PubMed Search performed on 14/03/2022

Search	Query
#4	**((#1) AND (#2)) AND (#3)**
#3	(Motor impairment[Title/Abstract]) OR (motor function[Title/Abstract])) OR (movement[Title/Abstract])) OR (motor test[Title/Abstract])) OR (motor dysfunction[Title/Abstract])) OR (Motor test[Title/Abstract]))) OR (fine motor test[Title/Abstract])) OR (motor performance[Title/Abstract])) OR (movement test[Title/Abstract])) OR (gross motor function[Title/Abstract])) OR (gross motor impairment[Title/Abstract])) OR (motor dysfunction[Title/Abstract])) OR (motor decline[Title/Abstract])) OR (finger tapping[Title/Abstract])) OR (apraxia[Title/Abstract])) OR (dyspraxia[Title/Abstract])) OR (dexterity[Title/Abstract])) OR (grasp*[Title/Abstract])) OR (grip*[Title/Abstract])) OR (tap*[Title/Abstract])) OR (hand tapping[Title/Abstract])) OR (keyboard tapping[Title/Abstract])) OR (holding[Title/Abstract])) OR (draw*[Title/Abstract])) OR (writ*[Title/Abstract])) OR (Purdue pegboard test[Title/Abstract])
#2	Hand*[Title/Abstract] OR Forearm*[Title/Abstract] OR Finger*[Title/Abstract] OR Upper limb*[Title/Abstract] OR "Upper limb"[Title/Abstract]
#1	Dementia*[Title/Abstract] OR "Cognitive impairment"[Title/Abstract] OR "Cognitive decline"[Title/Abstract] OR "Alzheimer’s Disease"[Title/Abstract] OR "Mild Cognitive Impairment"[Title/Abstract] OR Cognition*[Title/Abstract] OR "Cognitive domains"[Title/Abstract]
